# Klotho Pathways, Myelination Disorders, Neurodegenerative Diseases, and Epigenetic Drugs

**DOI:** 10.1089/biores.2020.0004

**Published:** 2020-03-31

**Authors:** Walter H. Moos, Douglas V. Faller, Ioannis P. Glavas, David N. Harpp, Iphigenia Kanara, Anastasios N. Mavrakis, Julie Pernokas, Mark Pernokas, Carl A. Pinkert, Whitney R. Powers, Konstantina Sampani, Kosta Steliou, Demetrios G. Vavvas, Robert J. Zamboni, Krishna Kodukula, Xiaohong Chen

**Affiliations:** ^1^ Department of Pharmaceutical Chemistry, School of Pharmacy, University of California San Francisco, San Francisco, San Francisco, California.; ^2^ ShangPharma Innovation, Inc., South San Francisco, California.; ^3^ Department of Medicine, Boston University School of Medicine, Boston, Massachusetts.; ^4^ Cancer Research Center, Boston University School of Medicine, Boston, Massachusetts.; ^5^ Department of Ophthalmology, New York University School of Medicine, New York, New York.; ^6^ Department of Chemistry, McGill University, Montreal, Canada.; ^7^ Hellenic Republic, Ministry of Foreign Affairs, Athens, Greece.; ^8^ Department of Medicine, Tufts University School of Medicine, St. Elizabeth's Medical Center, Boston, Massachusetts.; ^9^ Advanced Dental Associates of New England, Woburn, Massachusetts.; ^10^ Department of Pathobiology, College of Veterinary Medicine, Auburn University, Auburn, Alabama.; ^11^ Department of Health Sciences, Boston University, Boston, Massachusetts.; ^12^ Department of Anatomy, Boston University School of Medicine, Boston, Massachusetts.; ^13^ Department of Ophthalmology, Harvard Medical School, Boston, Massachusetts.; ^14^ Beetham Eye Institute, Joslin Diabetes Center, Boston, Massachusetts.; ^15^ PhenoMatriX, Inc., Natick, Massachusetts.; ^16^ Retina Service, Angiogenesis Laboratory, Massachusetts Eye and Ear Infirmary, Boston, Massachusetts.

**Keywords:** amyotrophic lateral sclerosis, Klotho, mitochondria, multiple sclerosis, neurodegenerative disease

## Abstract

In this review we outline a rationale for identifying neuroprotectants aimed at inducing endogenous Klotho activity and expression, which is epigenetic action, by definition. Such an approach should promote remyelination and/or stimulate myelin repair by acting on mitochondrial function, thereby heralding a life-saving path forward for patients suffering from neuroinflammatory diseases. Disorders of myelin in the nervous system damage the transmission of signals, resulting in loss of vision, motion, sensation, and other functions depending on the affected nerves, currently with no effective treatment. *Klotho* genes and their single-pass transmembrane Klotho proteins are powerful governors of the threads of life and death, true to the origin of their name, Fates, in Greek mythology. Among its many important functions, Klotho is an obligatory co-receptor that binds, activates, and/or potentiates critical fibroblast growth factor activity. Since the discovery of *Klotho* a little over two decades ago, it has become ever more apparent that when Klotho pathways go awry, oxidative stress and mitochondrial dysfunction take over, and age-related chronic disorders are likely to follow. The physiological consequences can be wide ranging, potentially wreaking havoc on the brain, eye, kidney, muscle, and more. Central nervous system disorders, neurodegenerative in nature, and especially those affecting the myelin sheath, represent worthy targets for advancing therapies that act upon Klotho pathways. Current drugs for these diseases, even therapeutics that are disease modifying rather than treating only the symptoms, leave much room for improvement. It is thus no wonder that this topic has caught the attention of biomedical researchers around the world.

## Background

The term “epigenetics” refers to changes resulting from modification of gene expression instead of alterations in the genetic code.^1^ We postulate that drugs aimed at inducing endogenous Klotho activity and expression—that is, therapeutics acting through epigenetic mechanisms—should promote remyelination and/or stimulate myelin repair by acting on mitochondrial function. As such, this approach may herald a life-saving path forward for patients suffering from neuroinflammatory diseases.

*Klotho*—a gene set of three members: *α-Klotho*, β-*Klotho*, and *γ-Klotho*^2–4^—is aptly named in the biological context of aging.^5,6^ According to Greek mythology, Klotho (or Clotho; Greek: Kλωθώ), the youngest of the Fates (Clotho, Lachesis: Λάχɛσις and Atropos: Άτροπος), is one of the three daughter deities (the spinner) of Zeus and Nyx (Nύξ, the goddess of night) or Themis (Θέμις, the goddess of law and order) who together spin out the thread of life, allot destiny, and choose the time of passing for both mortals and immortals.^7^ Thus, nothing could be more appropriate than *Klotho* serving as a longevity gene. Indeed, once *Klotho* fails to adequately express its proteins and variants,^6,8–10^ it is implicated in pathways that drive age-related chronic disorders such as kidney disease, tissue dysfunction, diabetic retinopathies, neurodegeneration, and impairments in mitochondrial function and muscle regeneration.^4,8,11–16^

α-Klotho is often referred to as an “anti-aging protein.”^3,6,17,18^ When overexpressed in mice, Klotho extends life (20–30%), reduces oxidative stress (OS), and demonstrates other prosurvival properties.^19–25^ The potential of extending these results to humans has captured pharmaceutical interest in developing Klotho-based therapeutics to hinder the degenerative illnesses of aging.^5,6,8,26–28^

Noticeably, a growing body of evidence asserts the therapeutic potential of Klotho in treating neurodegenerative diseases. As population aging is a global phenomenon,^29^ age-related neurodegenerative disorders are projected to surpass cancer as the foremost cause of death after cardiovascular disease in the developed world within 20 years.^30^ The late-onset sporadic form (LOAD) of Alzheimer's disease (AD)^31–33^ accounts for >90% of disease cases.^31,34–36^ Along with advanced aging,^23,37–43^ inheritance of the apolipoprotein E4 allele (also called *APOE4* or *APOEɛ4*) remains the most significant known genetic risk factor for LOAD. The risk is higher and the age at onset of dementia is younger for individuals carrying multiple copies of *APOE4*, whereas other *APOE* alleles are considered protective.^31,32,44^ In a study of a gene variant of *Klotho* with respect to AD in at-risk but presymptomatic individuals, heterozygosity was found to reduce amyloid aggregation in an *APOE4*-associated manner.^45^ Of interest, in a research analysis that measured Klotho concentrations in the cerebrospinal fluid of AD subjects and in older versus younger adults, Klotho levels were found to be lower in women compared with men.^46,47^ Perhaps the latter observation may help to explain why women are more likely than men to have AD, although the reported difference may be the result of biological or social artifacts.^48^ In addition to AD, the most common neurodegenerative disease, Parkinson's disease (PD), the second most common neurodegenerative disease,^49^ has also been tied to Klotho pathways.^50–52^

Beyond AD and PD, age-related declines in Klotho^8,13,17,24,53^ are associated with a range of other deteriorating central nervous system (CNS) processes.^17,24^ For example, mounting evidence implicates dysregulation of Klotho in shared mechanistic pathological relationships linking iron and myelin in various common and rare brain diseases,^54–56^ including abnormalities in myelination and the maturation of oligodendrocytes that are central to the pathogenicity of diseases such as multiple sclerosis (MS)^26,56,57^ and amyotrophic lateral sclerosis (ALS).^56,58^

## OS Demyelination and Mitochondrial Dysfunction

**FIG. 1.**
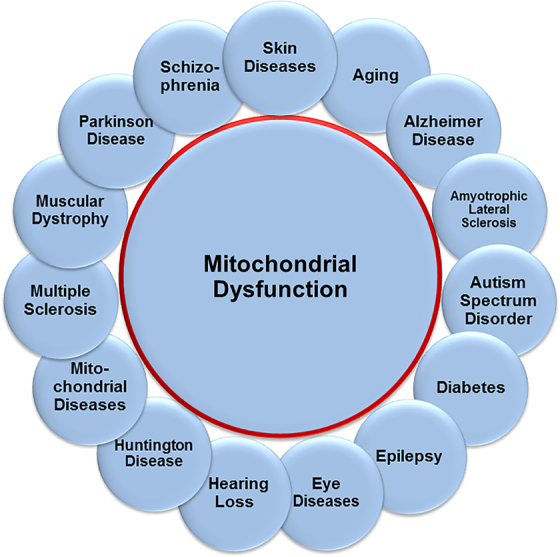
Sampling of neurologic conditions associated with mitochondrial dysfunction.

Mitochondrial dysfunction is a well-documented enabling factor in the pathophysiology of neurological conditions and disorders (Fig. 1).^41,59–68^ Although a principal role of mitochondria is to supply the bioenergy needed for cellular processes and maintenance,^69–71^ mitochondria also help regulate neurite branching and regeneration as well as synaptic strength, stability, and signaling in the CNS.^72^ In addition, myelin repair is intimately dependent on healthy mitochondrial function within the CNS in oligodendrocytes and neuronal cell bodies.^63,64,73–77^

Dysfunctional mitochondria become sources of reactive oxygen species (ROS) that contribute to OS with deleterious effects on the cell's well-being.^61,70,71,74,77–80^ Manifestations of OS are hallmark symptoms in neurological disease, including cognitive deficits.^52,54,65,76,79,81–88^ In concert with the above, a correlation was found between OS in the CNS and demyelination, which results in the loss of integrity and proper maintenance of oligodendrocytes and their myelin sheaths, the latter being crucial for cognitive performance and higher brain function.^57,89–91^

Thus, inclusion of strategies for enhancing mitochondrial biogenesis, function, and protection^68,80,92–96^ that may also rely on pathways epigenetically induced by diet^97–108^ and/or exercise^99,100,106–109^ can be timely in the therapeutic protocols for treating myelination disorders.^68,73–76,98,104,110–115^

## Dysregulated Myelination in Peripheral and CNS Diseases

Microglia are a distinct population of immune cells in the CNS.^116–118^ They execute fundamental tasks in brain development, physiology, and homeostasis and in influencing the pathological progression of brain diseases.^117–122^ There is evidence to suggest that microglia actively remove damaged myelin^114,123^ to recruit myelinating cells, oligodendrocytes in the CNS, and Schwann cells in the peripheral nervous system (PNS) to repair the injured myelin sheath.^114,117,118,123–125^ Dysregulated myelination is a characteristic feature of numerous heritable neurological diseases, such as the PNS hereditary disorder, Charcot–Marie–Tooth disease,^126,127^ X-linked adrenoleukodystrophy and metachromatic leukodystrophy,^128^ hereditary diffuse leukoencephalopathy with spheroids, Nasu–Hakola disease,^114^ and Huntington's disease,^129,130^ among others.^55,131,132^ A dysfunctional myelination apparatus is also evident in acquired demyelinating diseases such as diabetic peripheral neuropathy, drug-related peripheral neuropathies, leprosy, and peripheral neuropathies of inflammatory etiology.^132^

Most interestingly, converging evidence drawn from “Big Data” analytics in parallel with epigenetic, neuroimaging, and experimental model investigations seems to connect an adult-onset form of attention-deficit/hyperactivity disorder pathogenesis and persistence with dysregulated myelination.^133,134^ Many risk genes for CNS disorders such as AD, PD, schizophrenia, autism, and MS have been unveiled by genome-wide association studies to be expressed by microglia.^117^ Dysfunction of microglia is common in neurological diseases^114^ and recent studies have found that sex differences in microglial gene expression and functions seen in young adult mice tend to be increasingly pronounced in the aging brain.^135^

## Klotho as an Obligatory Co-receptor

High concentrations of phosphate in the body are found in bone, teeth, and dental enamel as calcium phosphate crystals.^136,137^ Klotho regulates phosphorus and calcium homeostasis ^5,6,18,23,138^ and functions as an obligatory co-receptor that binds and activates its related endocrine fibroblast growth factor (FGF) receptors (FGFRs) to potentiate its biological activities.^5,6,23,102,139–146^ FGFs are exemplary pleiotropic hormones that play numerous roles in cellular and metabolic homeostasis.^5,6,137,141,144–148^ In particular, FGF23 is a bone-derived hormone that in conjunction with Klotho acts on the kidney to increase phosphate excretion and suppress biosynthesis of vitamin D.^5,6,14,23,102,136,138,145,148,149^ Vitamin D regulates epigenetic mechanisms that maintain the transcription of its target genes in regulatory networks, including the expression of *Klotho* and nuclear factor-erythroid-2-related factor 2 (Nrf2) to carry out many of its homoeostatic functions.^17,97,150–153^ Vitamin D is a modulator of the immune system,^154,155^ hence its mention here, and accumulating evidence suggests vitamin D deficiency is a risk factor for dysregulated Klotho-associated neurodegenerative diseases, the most noteworthy being MS.^9,27,52,97,102,150,152,153,156–158^

## Multiple Sclerosis

MS is an insidious progressive neurodegenerative disease characterized by demyelinated lesions throughout the brain, spinal cord, and optic nerve resulting from immune-mediated attacks against myelin.^159–165^ It is the apotheosis of myelination disorders that affects ∼2.5 million people around the world^166–168^ and currently there are no definitive cures. The standard of chronic care, after using steroids for acute episodes, centers on the use of disease modifying therapies (DMTs) that modulate an overactive immune response, such as antibodies against interferon, interleukin, or related T cell targets.^9,169–172^ Unfortunately, although there is a growing armamentarium of DMTs for neurodegenerative diseases, they have to date had only a modest impact on disease progression^173,174^ and thus the demand for myelin repair-promoting therapies for MS remains a significant unmet medical need.^159,175–178^

**FIG. 2.**
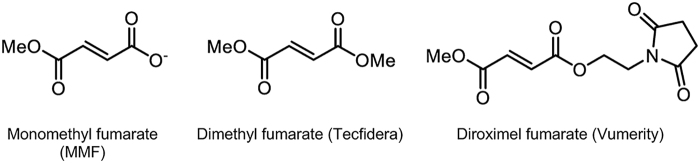
Monomethyl fumarate, the pharmacologically active form of Tecfidera and Vumerity.

The discovery of new drugs is a daunting, lengthy, and costly endeavor. Drug repurposing—based on mechanism of action and/or biological activity, not uncommonly the result of serendipity—is a promising and cost-saving approach for the treatment of rare genetic diseases and diseases with limited therapeutic options.^90,104,179,180^ This approach has yielded derivatives of the simple organic chemical, fumarate, including Vumerity (diroximel fumarate), which is reported to be better tolerated than Tecfidera, with fewer gastrointestinal side effects and more favorable pharmacokinetic properties. Vumerity is a delayed release formulation of an inactive diester prodrug of monomethyl fumarate (Fig. 2). Both Vumerity and Tecfidera are converted into the same pharmacologically active drug, monomethyl fumarate *in vivo*.^94,163^ The medical potential of dimethyl fumarate was identified over 60 years ago and marketed for the treatment of psoriasis.^181,182^ MS therapeutics approved by the U.S. Food and Drug Administration (FDA) are given in Table 1.

**Table 1.** Food and Drug Administration-Approved Drugs for Multiple Sclerosis in Disease Modifying Therapies

Older drugs, year approved	Recent approvals, year	Withdrawals, year
Betaseron (INF-β-1b), 1993	Lemtrada (alemtuzumab), 2014	Zinbryta (daclizumab), 2018
Avonex (INF-β-1a), 1996	Plegridty (INF-β-1a), 2014	
Copaxone (glatiramer acetate), 1996	Glatopa (glatiramer acetate), 2015	
Rebif (INF-β-1a), 2002	Ocrevus (ocrelizumab), 2017	
Tysabri (natalizumab), 2004	Mavenclad (cladribine), 2019	
Extavia (INF-β-1b), 2009	Mayzent (siponimod), 2019	
Gilenya (fingolimod), 2010	Vumerity (diroximel fumarate), 2019	
Aubagio (teriflunomide), 2012		
Tecfidera (dimethyl fumarate) 2013		

Sources: FDA Drug Approvals and Databases (www.fda.gov/drugs/development-approval-process-drugs/drug-approvals-and-databases). Orange Book: Approved Drug Products with Therapeutic Equivalence Evaluations (www.accessdata.fda.gov/scripts/cder/ob/index.cfm).

FDA, Food and Drug Administration.

## Klotho Structure, Distribution, and Function in MS

Klotho is a single-pass transmembrane protein expressed in the brain (hippocampus and choroid plexus), kidney, eye (retina, optic nerve, lens) and parathyroid gland, and less so in other tissues.^3,18,27,102,183,184^ A soluble form of Klotho (sKlotho), primarily secreted from the kidney, circulates in blood, urine, and cerebrospinal fluid, exerting different biological effects in multiple tissues as a humoral factor.^5,6,8,52,185–189^

In the eye, *Klotho* protects against OS^53,55,58,153,190,191^ and is essential to the proper maintenance and function of the ocular system,^12,26,192–195^ being expressed throughout the retina, with the highest levels in retinal ganglion cells.^196^ The retinal pigment epithelium (RPE) is a highly specialized CNS tissue whose function is critical in preserving retinal homeostasis^53,78^ and an age-dependent decline of *Klotho* expression is said to contribute to RPE degeneration and retinal pathology.^53^ Apoptotic cells in models of retinal degeneration were found to exhibit high levels of Klotho,^8^ which is consistent with Klotho overexpression in its role as a protective protein that inhibits apoptosis.^22,197,198^ A recent study has shown that higher levels of circulating Klotho protein is protective in patients with diabetic retinopathy.^199^

Although the retina itself is a nonmyelinated tissue,^200^ optic neuritis, a disease affecting the myelinated part of retinal ganglion cell axons, is a serious and often difficult to assess manifestation of MS,^201^ particularly in the pediatric population.^202^ More than 70% of MS patients suffer vision loss as a secondary effect of optic neuritis disease progression.^160,203,204^ In recent studies, Klotho was shown to accelerate remyelination in a cuprizone-mediated demyelination mouse model.^9,28^ This important finding is refocusing attention on Klotho's role in neurodegeneration and research efforts are increasingly directed toward the development of MS treatments that promote remyelination and/or stimulate myelin repair.^9,27,28,112,166,169,205–211^ However, because Klotho does not cross the blood–brain barrier,^10,212^ a small molecule approach aimed at inducing endogenous Klotho activity and expression in the CNS is surfacing as a promising therapeutic strategy.^27,81,90,143,213,214^ Epigenetics^10,90,100,169,215–219^ and gene therapy-based methods are part of the emerging landscape under investigation.^19,215,220,221^

## Amyotrophic Lateral Sclerosis

The global prevalence of ALS is estimated to be roughly two to four cases per 100,000 population^222,223^ compared with ∼30 cases per 100,000 population for MS.^168^ ALS (also referred to as progressive muscular atrophy or Lou Gehrig's disease) is a devastating neurodegenerative disease. It damages motor neurons in the brain and spinal cord leading to progressive muscle atrophy and paralysis that is fatal, usually within 3–5 years of diagnosis.^58,224–227^ Unfortunately, patients with ALS, at present, have limited therapeutic options (Table 2).^96,173,228^ Moreover, given the rapid and terminal progression of the disease postdiagnosis, there is a pressing need to develop new therapies and/or based on mechanism of action repurposing drugs already approved for other diseases.^176,180^ Recruiting ALS subjects into traditional clinical trials is challenging because of the low number of cases in the population. Trial-design protocols^229^ that rely on restrictive inclusion criteria, frequent study visits, use of a placebo control arm that denies patients early access to the therapy, and the comparatively long time it takes to document results relative to the rapid progression of the disease are additional impediments.^230^

**Table 2.** Food and Drug Administration-Approved Drugs for Treating Amyotrophic Lateral Sclerosis

Glutamate antagonist	Antioxidant	Other drugs
Riluzole (Rilutek/Teglutik)	Edaravone (Radicava/Radicut)	Dextromethorphan hydrobromide/quinidine sulfate (Neudexta) for pseudobulbar affect

Sources: FDA Drug Approvals and Databases (www.fda.gov/drugs/development-approval-process-drugs/drug-approvals-and-databases). Orange Book: Approved Drug Products with Therapeutic Equivalence Evaluations (www.accessdata.fda.gov/scripts/cder/ob/index.cfm).

**FIG. 3.**
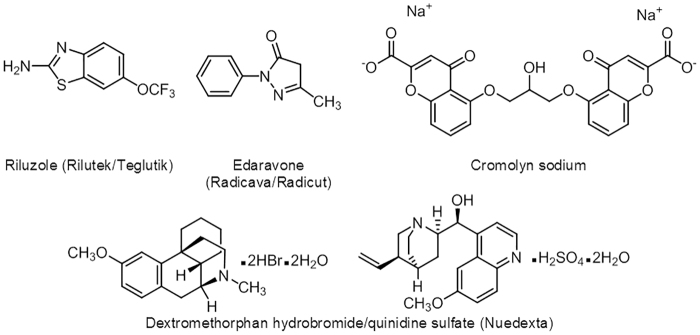
Chemical structures of FDA-approved therapeutics for ALS, including cromolyn sodium, a drug used to treat asthma and other conditions showing promising potential as a repurposed drug for ALS. ALS, amyotrophic lateral sclerosis; FDA, Food and Drug Administration.

Riluzole and edaravone, the principal therapeutics used in the treatment of ALS (Fig. 3), have a modest impact on disease progression, extending survival by ∼3 months.^68,96,223,231^ The combination of dextromethorphan and quinidine sulfate has shown positive results against pseudobulbar affect (emotional lability) and is FDA-approved for ALS and MS,^232^ although it is reported to be prescribed more to patients suffering from dementia or PD.^233^ Clearly, much more effective therapies are needed and a vigorous research effort has been underway for the past several years to screen for and develop new pharmaceuticals for treating neurodegenerative diseases including ALS.^234,235^ Cromolyn sodium (Fig. 3), an FDA-approved compound used to treat asthma and other conditions has recently emerged as a promising new therapeutic for ALS. In the SOD1^G93A^ mouse model of ALS, treatment with cromolyn sodium delayed disease onset and showed neuroprotection by decreasing the inflammatory response.^236^ However, a focus on myelination may lead to more lasting and effective therapeutic outcomes. Klotho overexpression in the SOD1^G93A^ mouse model was shown to suppress the production of proinflammatory cytokines, reduce the expression of neuroinflammatory markers, and prevent neuronal loss with a more profound effect in the spinal cord than in the motor cortex, thereby delaying the onset and progression of the disease.^58^ These results along with the positive effect Klotho has on the promyelinating properties of oligodendrocytes offer compelling evidence in support of developing Klotho-based therapeutic strategies for treating ALS.^58^

## Concluding Remarks

**FIG. 4.**
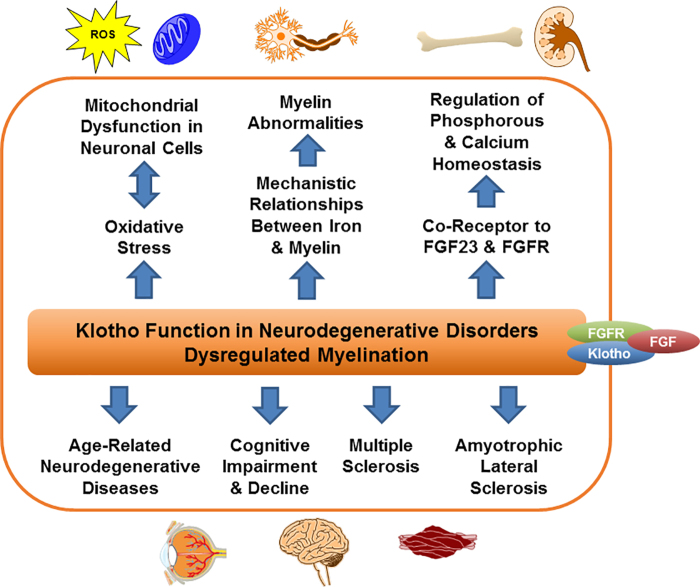
Klotho function in neurodegenerative disorders and/or dysregulated myelination.

As outlined in the introduction, drugs aimed at inducing endogenous Klotho activity and expression—epigenetic action *per se*—should promote remyelination and/or stimulate myelin repair by acting on mitochondrial function. In the ensuing two decades since the serendipitous discovery of *Klotho* as an aging-suppressor gene, research has helped unmask many of its functional pathways in neurodegenerative disorders and/or dysregulated myelination (Fig. 4). Deficient levels of Klotho protein lead to excessive OS induction mainly from ROS produced in mitochondrial dysfunction. Myelin repair is intimately dependent on the energy made available by healthy mitochondria within the CNS in oligodendrocytes (Schwann cells in the PNS) and neuronal cell bodies. Thus, drugs aimed at inducing endogenous Klotho production may herald a life-saving path forward for patients suffering from neuroinflammatory diseases. In parallel, much as the old psoriasis drug, dimethyl fumarate, was repurposed to treat MS, more drug repurposing may find worthwhile paths here too. Will AD, PD, MS, or ALS yield to these approaches when coupled with drugs that attack such a powerful pathway as Klotho? As we kick off what we hope will be “the roaring 2020s” when it comes to the advancement of major new life-saving therapeutics, time and effort toward this goal will hopefully give us the answers.

## Abbreviations Used

AD: Alzheimer's disease
ALS: amyotrophic lateral sclerosis
CNS: central nervous system
DMT: disease modifying therapy
FDA: Food and Drug Administration
FGF: fibroblast growth factor
LOAD: late-onset Alzheimer's disease
MS: multiple sclerosis
OS: oxidative stress
PD: Parkinson's disease
PNS: peripheral nervous system
ROS: reactive oxygen species
